# Are Probiotics Beneficial or Harmful for Pancreatic Cancer Outcomes?

**DOI:** 10.1007/s12602-024-10437-7

**Published:** 2024-12-23

**Authors:** Tae Seung Lee

**Affiliations:** Division of Gastroenterology, Department of Internal Medicine and Liver Research Institute, Seoul National University Hospital, Seoul National University College of Medicine, 101, Daehak-Ro, Jongno-Gu, Seoul, Republic of Korea

**Keywords:** Probiotics, Tumor microbiome, Pancreatic cancer

## Abstract

Pancreatic cancer is influenced by interactions between cancer cells and the tumor microenvironment (TME), including tumor-infiltrating lymphocytes (TILs). Specifically, CD8 + T cells impact prognosis by eliminating cancer cells. Recent studies have revealed that microbiomes are present in pancreatic tissues and may affect tumor growth and immune responses. Additionally, recent studies revealed that the abundance of *Bacteroides*, *Lactobacillus*, and *Peptoniphilus* are associated with poor pancreatic cancer prognosis. This study investigates the role of oral probiotics in influencing pancreatic cancer outcomes. We retrospectively reviewed patients aged ≥ 18 years with pathologically confirmed pancreatic cancer from Seoul National University Hospital between January 2011 and January 2023. We investigated progression-free survival and overall survival between the control group and the probiotics group. Among pancreatic cancer patients undergoing palliative chemotherapy without radiotherapy and resection, there was a significant difference in overall survival (OS) when comparing the control group to the probiotics group (median: 10 months (9–11) vs. 12 months (9–19), *p* = 0.026). Regardless of the type of probiotics, oral probiotics may have a positive impact, but further research is still needed to understand the underlying immunological mechanisms.

## Introduction

Pancreatic cancer is known to be one of the most challenging cancers with a poor prognosis. Surgical resection is the only curative option; however, less than 20% of patients are suitable for surgical resection at diagnosis, leading to a 5-year survival rate of approximately 11%. At the time of diagnosis, about 50–60% of patients are found to have metastasized, 25–30% have locally advanced disease, and only 10–15% of patients are eligible for surgery with localized disease at the time of diagnosis. Even after surgery, recurrence is very frequent [1]. Furthermore, the incidence of pancreatic cancer is increasing by 0.5–1% annually not only in the United States but also in other countries. By 2030, pancreatic cancer is predicted to become the second leading cause of cancer-related deaths [2].

Despite the development of several standard treatments, the 5-year survival rate for pancreatic cancer remains low [3–6]. This is because the tumor microenvironment, which includes cancer-associated fibroblasts (CAFs) and tumor-infiltrating lymphocytes (TILs), is well-developed, leading to poor responses to chemotherapy [7–9]. Tumor-infiltrating CD8 + T cells affect pancreatic cancer outcomes by targeting cancer cells [10]. Although pancreatic tissue was thought to be sterile, recent studies using mouse models have shown that microbiomes from the oral cavity or duodenum can migrate to the pancreas [11]. Research has also suggested that the presence of intratumoral microbiome in pancreatic cancer is related to tumor development and prognosis through the suppression of both innate and adaptive immune responses [12, 13]. According to a recent study, the presence of intratumoral anaerobic microbiomes such as *Bacteroides*, *Lactobacillus*, and *Peptoniphilus* is associated with a decrease in CD4 + , CD8 + , and CD45RO + T cells and poor prognosis in pancreatic cancer [14].

However, *Lactobacillus* is commonly used as a probiotic ingredient, often prescribed in clinical settings or purchased over the counter for regular consumption [15, 16]. While previous studies have reported the presence of intratumoral *Lactobacillus* with poor prognosis [14], however, some studies have suggested that externally introduced *Lactobacillus* species may have a mitigating effect on pancreatic cancer [17, 18]. It remains controversial whether the actual intake of such probiotics is directly associated with poor outcomes in pancreatic cancer. Therefore, this study aims to investigate the relationship between exposure to oral probiotics and the outcomes of pancreatic cancer in the real world.

## Methods

### Study Population

Patients aged ≥ 18 years diagnosed with pathologically confirmed pancreatic cancer at Seoul National University Hospital from January 2011 to January 2023 were enrolled in this study. Patients diagnosed with pancreatic cancer and treated with chemotherapy were enrolled, specifically those who experienced progression during curative treatment or were diagnosed with metastatic disease, and were either undergoing or planning to undergo palliative chemotherapy. The retrospective review was performed regardless of whether the patients underwent surgery or radiation therapy. All prescriptions for probiotics, such as *Lactobacillus rhamnosus/Lactobacillus helveticus* 20 mg, *Lactobacillus casei/Lactobacillus rhamnosus* 250 mg, and *Saccharomyces boulardii* 250 mg, up to the last follow-up date were reviewed, regardless of the cancer diagnosis. Probiotics were prescribed for gastrointestinal symptoms such as vomiting, nausea, diarrhea, or constipation when there was an indication and the clinician deemed their use necessary. Clinical and demographic parameters were retrospectively reviewed from electronic medical records, including age, sex, date of the first imaging diagnosis of pancreatic cancer, tumor location and size, date of resection, the initial and last dates of palliative chemotherapy, and the type of chemotherapy.

### Study Outcomes

The primary outcome was progression-free survival, and the secondary outcome was overall survival in propensity score-matched patients. Progression-free survival (PFS) and overall survival (OS) were calculated from the date of initial palliative chemotherapy. Progression was defined based on the date when a newly appeared lesion or recurrence was first detected on CT or imaging according to the Response Evaluation Criteria in Solid Tumors (RECIST) guideline (version 1.1). Death was recorded based on documented evidence or, in the absence of documentation, verified through mortality data. In cases of hopeless discharge or hopeless follow-up loss, the last follow-up date was considered the date of death.

### Statistical Analysis

The *χ*^2^ and Fisher’s exact tests were used to compare qualitative variables. The Shapiro–Wilk test was used to assess normality for quantitative variables. Student’s *t*-test was used to analyze continuous variables with normal distribution; the Mann–Whitney *U*-test was performed otherwise. Categorical variables were assessed with the chi-square test or Fisher’s exact test. A *p*-value of ≤ 0.05 indicated significant differences, and the respective variables were regarded as covariates for adjustment in each analysis. Furthermore, 95% confidence intervals (CIs) were provided for PFS and OS, which were estimated using the Kaplan–Meier method and reported as median and range. To estimate the propensity score, logistic regression was used with the following covariates: age, sex, conversion to curative resection, and radiotherapy. We matched the two groups of patients using a one-to-one nearest-neighbor matching protocol.

## Results

### Clinical Characteristics of the Study Population

Of the 632 patients, the 569 who had never been prescribed probiotics were categorized as the control group, while the 63 patients who had been prescribed probiotics were classified as the probiotics group. There were no differences between the two groups in terms of age, sex, tumor size, tumor location, presence of metastasis, curative resection before chemotherapy, and no evidence of disease (NED) status. However, differences were observed between the two groups in terms of conversion to curative resection status, radiotherapy, and follow-up duration. After performing propensity score matching, the standard mean difference for the overall distance was 0.0023, for age was − 0.0521, for sex was 0.0320, for conversion to resection status was < 0.0001, for radiotherapy status was < 0.0001, and for the number of lines of chemotherapy was < 0.0001. These results confirmed that the overall distance values were below 0.1, indicating a relatively well-balanced comparison between the two groups. There were no significant differences in baseline characteristics between the two groups (Table 1). The prescription rate for probiotics was 1.6% for *Lactobacillus rhamnosus/Lactobacillus helveticus* (1 patient), 42.6% for *Saccharomyces boulardii*, and 62.3% for *Lactobacillus casei* and *L. rhamnosus* (Table 2).

**Table 1:** Baseline characteristics of the study patients^*^

	All patients			After propensity score matching		
	Control	Probiotics	*p*	Control	Probiotics	*p*
	(*N* = 569)	(*N* = 63)		(*N* = 63)	(*N* = 63)	
Sex			0.832			0.856
Male	347 (61.0%)	37 (58.7%)		39 (61.9%)	37 (58.7%)	
Female	222 (39.0%)	26 (41.3%)		24 (38.1%)	26 (41.3%)	
Age	61.4 ± 9.7	60.8 ± 11.3	0.623	61.2 ± 10.2	60.8 ± 11.3	0.830
Tumor size (cm)	3.8 ± 1.9	3.8 ± 1.7	0.780	3.9 ± 1.6	3.8 ± 1.7	0.530
Tumor location			0.804			0.973
Head	229 (40.2%)	26 (41.3%)		26 (41.3%)	26 (41.3%)	
Body	166 (29.2%)	16 (25.4%)		17 (27.0%)	16 (25.4%)	
Tail	174 (30.6%)	21 (33.3%)		20 (31.7%)	21 (33.3%)	
Metastasis	299 (52.5%)	37 (58.7%)	0.424	39 (61.9%)	37 (58.7%)	0.856
Pre-chemotherapy resection	101 (17.8%)	17 (27.0%)	0.106	11 (17.5%)	17 (27.0%)	0.284
Conversion to resection	22 (3.9%)	9 (14.3%)	0.001	8 (12.7%)	9 (14.3%)	> 0.999
Radiotherapy	102 (17.9%)	20 (31.7%)	0.014	21 (33.3%)	20 (31.7%)	> 0.999
The number line of palliative chemotherapy^†^	2.1 ± 1.0	2.5 ± 1.0	0.011	2.5 ± 1.0	2.5 ± 1.0	> 0.999
Progression	491 (86.3%)	51 (81.0%)	0.337	59 (93.7%)	51 (81.0%)	0.061
Death	557 (97.9%)	58 (92.1%)	0.021	61 (96.8%)	58 (92.1%)	0.437
NED^‡^ state	4 (0.7%)	1 (1.6%)	0.998	2 (3.2%)	1 (1.6%)	> 0.999
Follow-up duration (months)	14.3 ± 14.8	22.1 ± 20.8	0.005	16.2 ± 15.6	22.1 ± 20.8	0.073

^*^Values are presented as mean ± standard deviation. Other values are presented as number (%)

^†^The chemotherapy regimen distribution across all cycles was as follows: In the control group, 5-FU-based was 531 (18.7%), gemcitabine-based was 432 (15.2%), S-1 or capecitabine was 91 (3.2%), and trial was 8 (0.3%). In the probiotics group, 5-FU-based was 79 (25.1%), gemcitabine-based was 56 (17.8%), S-1 or capecitabine was 10 (3.2%), and trial was 3 (1.0%)

^‡^*NED*, no evidence of disease

**Table 2:** Type of probiotics strains

Type of probiotics	No. (%)
Lactobacillus rhamnosus/Lactobacillus helveticus	1 (1.6%)
Saccharomyces boulardii	26 (42.6%)
Lactobacillus casei/Rhamnosus species	38 (62.3%)

### Primary and Secondary Outcomes

**Fig. 1:**
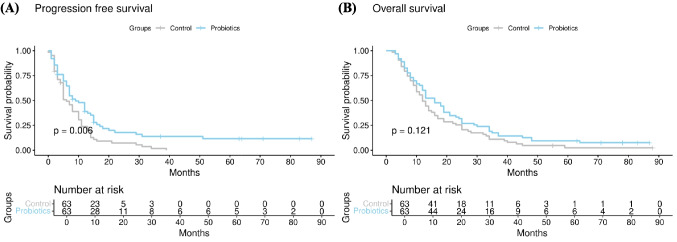
Comparison of **A** progression-free survival and **B** overall survival in propensity score-matched patients

There was a significant difference in PFS when comparing the control group with the probiotics group (median, 6 months (5–10) vs. 9 months (7–14), *p* = 0.006) (Fig. 1A). There was a significant difference in OS when comparing the control group with the probiotics group (median, 12 months (10–16) vs. 16 months (12–21), *p* = 0.121) (Fig. 1B).

### Comparison of Survival Rates in Patients Who Did Not Undergo Curative Resection

**Fig. 2:**
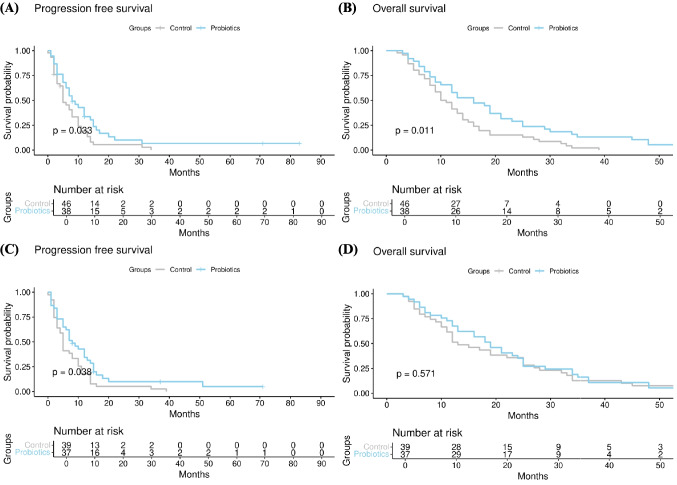
**A** Progression-free survival (PFS) and **B** overall survival (OS) in propensity score-matched patients without curative resection. **C** Progression-free survival (PFS) and **D** overall survival (OS) in propensity score-matched patients with metastatic disease

When comparing patients who were unable to undergo curative surgery or either before or after chemotherapy in propensity score-matched patients, there was a significant difference in PFS between the control group and the probiotics group (median, 5 months (5–10) vs. 8 months (6–15), *p* = 0.033) (Fig. 2A). Additionally, There was a significant difference in OS when comparing the control group with the probiotics group (median, 10.5 months (9–14) vs. 16 months (12–23), *p* = 0.011) (Fig. 2B).

### Comparison of Survival Rates in Patients with Unresectable Disease

When comparing patients with metastatic disease, there was a significant difference in PFS between the control group and the probiotics group (median, 5 months (4–10) vs. 8 months (6–14), *p* = 0.038) (Fig. 2C). However, There was not a significant difference in OS when comparing the control group with the probiotics group (median, 13 months (11–25) vs. 19 months (13–25), *p* = 0.571) (Fig. 2D).

### Comparison of Survival Rates Based on the Type of Probiotics

**Fig. 3:**
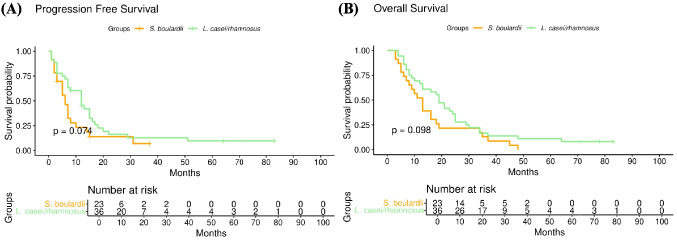
Comparison of survival rates based on the type of probiotics

Excluding the patient who received *Lactobacillus rhamnosus/Lactobacillus helveticus* and those who were administered both *Saccharomyces boulardii* and *Lactobacillus casei*/*Lactobacillus rhamnosus*, there were no significant differences in PFS (median, 6 months (5–10) vs. 12 months (12–23), *p* = 0.074) and OS (median, 13 months (8–19) vs. 19 months (13–25), *p* = 0.098) based on the type of probiotics (Fig. 3).

### Multivariate Cox Regression Analysis for PFS and OS

**Fig. 4:**
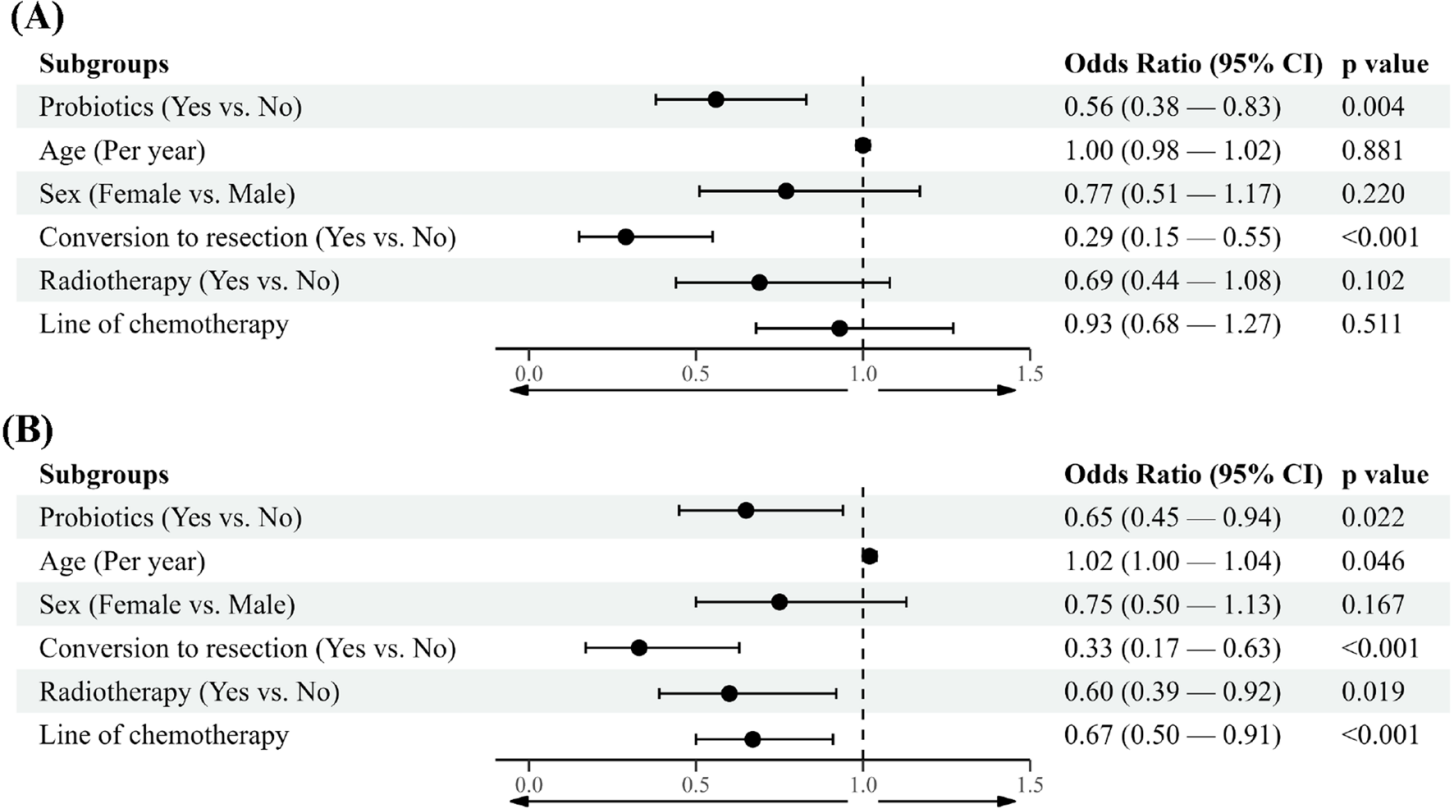
Multivariate Cox regression analysis for **A** progression-free survival and **B** overall survival

We conducted a multivariate Cox regression analysis, adjusting for variables such as age, sex, conversion to curative resection, and the number of lines of palliative chemotherapy (Table 3). In terms of progression-free survival, probiotic exposure (HR, 0.56 (CI, 0.38–0.83), *p* = 0.004) and the presence of curative surgery either before or after chemotherapy showed statistically significant associations (HR, 0.29 (CI, 0.15–0.55), *p* < 0.001). In terms of overall survival, probiotic exposure (HR, 0.65 (CI, 0.45–0.94), *p* = 0.022), the presence of curative surgery either before or after chemotherapy (HR, 0.33 (CI, 0.17–0.63), *p* < 0.001), radiotherapy (HR, 0.60 (CI, 0.39–0.92), *p* = 0.019), and a higher number of lines of chemotherapy (HR, 0.67 (CI, 0.50–0.91), *p* < 0.001) were found to be associated with a lower hazard ratio. Older age (HR, 1.02 (CI, 1.00–1.04), *p* = 0.046) was found to be associated with a higher hazard ratio for overall survival (Fig. 4).

**Table 3:** Univariate Cox regression analysis for (A) progression-free survival and (B) overall survival

	PFS	*p*-value	OS	*p*-value
	Hazard ratio (95% Cl)		Hazard ratio (95% Cl)	
Probiotics (yes vs. no)	0.59 (0.40–0.86)	0.010	0.76 (0.53–1.09)	0.130
Age (per year)	1.01 (0.99–1.03)	0.210	1.02 (1.01–1.04)	0.010
Sex (female vs. male)	0.92 (0.62–1.35)	0.660	0.88 (0.61–1.28)	0.500
Tumor size (per centimeter)	1.00 (0.89–1.11)	0.940	0.99 (0.89–1.10)	0.810
Tumor location	0.77 (0.61–0.97)	0.020	0.79 (0.64–0.98)	0.030
Metastatis (yes vs. no)	1.58 (1.06–2.35)	0.020	0.77 (0.53–1.12)	0.170
Pre-chemotherapy resection	1.49 (0.96–2.33)	0.080	1.12 (0.72–1.72)	0.620
Conversion to resection	0.27 (0.14–0.50)	< 0.001	0.24 (0.13–0.45)	< 0.001
Radiotherapy	0.65 (0.44–0.97)	0.040	0.54 (0.37–0.80)	< 0.001
Total line of chemotherapy	0.83 (0.68–1.01)	0.070	0.61 (0.50–0.75)	< 0.001
Follow-up duration (per month)	0.92 (0.90–0.94)	< 0.001	0.74 (0.70–0.77)	< 0.001

## Discussion

Some *Lactobacillus species* and *Saccharomyces species* are known to potentially aid in the treatment of colorectal cancer [19–21], and *Lactobacillus species* may also be beneficial in hepatocellular carcinoma in cases of type 2 diabetes mellitus [22]. Additionally, *Lactobacillus species* are also known to influence immunopathogenesis in endometrial cancer [23]. A study reported that the dietary tryptophan metabolite indole-3-aldehyde, produced by intratumoral *Lactobacillus reuteri*, promotes immune checkpoint inhibitor (ICI) efficacy and survival in advanced melanoma patients [24]. One study reported that the supernatant of *Saccharomyces boulardii* may contribute to the anticancer effect against breast cancer cells [21].

However, regarding esophageal cancer, there are reports suggesting that the intratumoral microbiome and microbiome alpha-diversity may actually influence immune infiltration in the tumor microenvironment, potentially being associated with poor prognosis [25]. In pancreatic cancer as well, there are reports indicating that the intratumoral microbiome and microbiome alpha-diversity are associated with poor prognosis [14]. Despite this, there are also reports suggesting that externally derived *Lactobacillus* may have beneficial effects on prognosis, as mentioned earlier [17, 18].

This study investigated the impact of commonly prescribed probiotics in Korea on patients with pancreatic cancer, aligning with previous findings that *Lactobacillus* species, whether co-cultured [17] or administered orally [18], are associated with beneficial outcomes. Considering that humans develop immune tolerance towards gut commensal microbiota and that immunity develops in conjunction with gut microbiota [26], these results suggest that externally derived microbiomes may influence immunity differently from intratumoral microbiomes, potentially affecting cancer prognosis. Therefore, additional research is needed to understand the immunological mechanisms by which both intratumoral and external microbiomes influence cancer prognosis.

In our study, after propensity score matching, no difference in overall survival was observed between the probiotics group and the control group in the overall population. However, a difference was noted among patients who were unable to undergo curative resection, while no such difference was observed in patients with advanced-stage metastatic disease. While surgery provides a survival benefit in pancreatic cancer [27], our findings suggest that probiotics may potentially offer a survival benefit in early-stage patients with borderline resectable or locally advanced pancreatic cancer who are unable to undergo surgery. Additionally, the small number of patients in the probiotics group is likely due to the fact that probiotics are not typically prescribed for pancreatic cancer patients.

Our study has the following limitations: (1) Since the investigation was conducted retrospectively by reviewing prescription records, it does not fully reflect the actual intake of probiotics. Specifically, probiotics can be purchased over the counter in addition to being prescribed. Moreover, as many commercial beverages contain added probiotics, the duration, type, and dosage of probiotics to which patients were exposed were not accounted for in this study. Further randomized controlled trials (RCTs) will be necessary and important in the future. (2) The mechanism of how the exposed probiotics affect the TME and intratumoral microbiome was not elucidated. (3) There were not enough patients who took probiotics compared to those who did not.

However, our study is significant in that it enrolled a large number of patients and investigated real-world oral probiotic use to analyze pancreatic cancer prognosis. Our results suggest that oral probiotics may have a positive impact, but further research is still needed to understand the underlying immunological mechanisms.

## Conclusion

Regardless of the type of probiotics, it was found that probiotics exposure may positively affect the survival of pancreatic cancer patients in a palliative setting.

## Data Availability

No datasets were generated or analysed during the current study.
